# Cannabinoid CB2 Receptor Mediates Nicotine-Induced Anti-Inflammation in N9 Microglial Cells Exposed to *β* Amyloid via Protein Kinase C

**DOI:** 10.1155/2016/4854378

**Published:** 2016-01-13

**Authors:** Ji Jia, Jie Peng, Zhaoju Li, Youping Wu, Qunlin Wu, Weifeng Tu, Mingchun Wu

**Affiliations:** ^1^Department of Anesthesiology, Guangzhou General Hospital of Guangzhou Military Command, Guangzhou 510010, China; ^2^Department of Anesthesiology, Wuhan General Hospital of Guangzhou Military Command, Wuhan 430070, China

## Abstract

*Background*. Reducing *β* amyloid- (A*β*-) induced microglial activation is considered to be effective in treating Alzheimer's disease (AD). Nicotine attenuates A*β*-induced microglial activation; the mechanism, however, is still elusive. Microglia could be activated into classic activated state (M1 state) or alternative activated state (M2 state); the former is cytotoxic and the latter is neurotrophic. In this investigation, we hypothesized that nicotine attenuates A*β*-induced microglial activation by shifting microglial M1 to M2 state, and cannabinoid CB2 receptor and protein kinase C mediate the process.* Methods*. We used A*β*1–42 to activate N9 microglial cells and observed nicotine-induced effects on microglial M1 and M2 biomarkers by using western blot, immunocytochemistry, and enzyme-linked immunosorbent assay (ELISA).* Results*. We found that nicotine reduced the levels of M1 state markers, including inducible nitric oxide synthase (iNOS) expression and tumor necrosis factor *α* (TNF-*α*) and interleukin- (IL-) 6 releases; meanwhile, it increased the levels of M2 state markers, including arginase-1 (Arg-1) expression and brain-derived neurotrophic factor (BDNF) release, in the A*β*-stimulated microglia. Coadministration of cannabinoid CB2 receptor antagonist or protein kinase C (PKC) inhibitor partially abolished the nicotine-induced effects.* Conclusion*. These findings indicated that cannabinoid CB2 receptor mediates nicotine-induced anti-inflammation in microglia exposed to A*β* via PKC.

## 1. Introduction

*β* amyloid- (A*β*-) induced microglial activation plays a vital role in the pathogenesis of Alzheimer's disease (AD) [1, 2]. A*β* binds to microglia and activates them to secrete inflammatory cytokines, including tumor necrosis factor *α* (TNF-*α*), interleukin- (IL-) 1*β* and IL-6 [3]. Long-term inflammation in brain induces neuronal cell injury and even cell death [4]. Some latest investigations suggested that microglia can be activated into classic activated state (M1 state) or selective activated state (M2 state) [5–7]. In the M1 state, activated microglial cells produce proinflammatory cytokines, which are considered to be detrimental; in contrast, activated microglial cells of M2 state secrete anti-inflammatory cytokines and neurotrophins, including IL-10, brain-derived neurotrophic factor (BDNF), and glial cell-derived neurotrophic factor (GDNF), which are regarded to be beneficial. Therefore, shifting microglial M1 to M2 state is considered to be an effective therapy for AD [8].

Nicotine is a main constituent of tobacco, which induces neuroprotection against A*β* both in vivo and in vitro [9, 10]. In addition, some studies showed that nicotine attenuates A*β*-induced microglial activation [11, 12]. However, the mechanism of nicotine-induced neuroprotective effect against AD is still not clear. Traditionally, nicotinic acetylcholine receptor (nAchR) is believed to be involved in the nicotine-induced protection against A*β* in neurons and microglia [12, 13]. Yet emerging evidences support that there might be a cross-talk between nicotine administration and the endocannabinoid system. It has been found that cannabinoid CB2 receptor antagonist is used to treat nicotine addiction [14], and CB2 receptor plays a relevant role in the rewarding, reinforcing [15], and motivational effects of nicotine [16]. Moreover, cannabis tetrahydrocannabinol (THC) reduces the incidence of precipitated nicotine withdrawal signs in mice [17]. In addition, we have ever reported that cannabinoid CB1 receptor (CB1) is involved in nicotine-induced neuroprotection against A*β* in neurons, and protein kinase C (PKC) mediates the protection [18]. It is proved that cannabinoid CB2 receptor (CB2) is expressed in microglial cells, and we have ever reported that upregulation of CB2 receptor shifts microglial M1 to M2 state, leading to neuroprotective effects [19]. However, whether CB2 receptor is involved in nicotine-induced anti-inflammation in microglial cells has not been reported.

In this study, we used microglial cells exposed to A*β* to mimic the neuroinflammation of AD and hypothesized that CB2 receptor mediates nicotine-induced anti-inflammation in A*β*-treated microglia by shifting microglial M1 to M2 state, and PKC may play a role in the anti-inflammation.

## 2. Materials and Methods

### 2.1. Materials

The N9 microglial cell-line was a gift from the Chinese Academy of Sciences. The nicotine, *β* amyloid 1–42 (A*β*), Iscove's modified Dulbecco's medium (IMDM), fetal bovine serum (FBS), and chelerythrine (protein kinase C inhibitor) were purchased from Sigma-Aldrich (St. Louis, MO, USA). CB2 receptor antagonist AM630 was purchased from Alexis Biochemicals (CH). The primary anti-CB2 and anti-PKC antibodies were purchased from Abcam Ltd. (Cambridge, UK). The anti-iNOS and anti-Arg-1 antibodies were purchased from Chemicon (USA). Bovine serum albumin (BSA), Cy3-labeled secondary antibody, FITC-labeled secondary antibody, anti-GAPDH antibody, and secondary horseradish peroxidase-conjugated goat anti-rabbit antibody were purchased from Beijing Cowin Bioscience Co., Ltd. (China). The DAPI staining solution was purchased from Beyotime (China). The enzyme-linked immunosorbent assay (ELISA) kits were purchased from PeproTech Inc. (USA).

### 2.2. Cell Culture

The N9 microglial cells were cultured in the IMDM medium containing 5% FBS, 100 U/mL penicillin, 100 *μ*g/mL streptomycin, and 2 mM glutamine. The humidified atmosphere of the cell culture incubator consisted of 95% air and 5% CO_2_. The medium was changed every 3 days. The cells were passaged 2-3 times per week and used within 8 weeks.

### 2.3. Subcellular Fractionation of Proteins and Western Blot Analysis

N9 microglial cells were seeded into a 6-well plate at a density of 2 × 10^5^ cells/well. After the treatments, the cells were homogenized on ice with the lysis buffer (0.3 M sucrose, 0.15 M NaCl, 20 mM Tris-HCl, 2 mM EDTA, 0.3 mM PMSF, and 10 *μ*g/mL leupeptin). Homogenates were centrifuged at 1000 ×g for 10 min at 4°C, and supernatant fractions were collected for ultracentrifugation. Cytosol and membrane fractions were separated by ultracentrifugation at 100,000 ×g for 90 min at 4°C. The supernatant constituted the cytosol fraction, and the pellet was resuspended and homogenized in the above lysis buffer with 0.2% Triton X-100 added. This resuspended fraction represented the membrane fraction. The Bradford reagent was used to compare PKC concentration in each fraction, and 20 *μ*g of total lysate from each fraction was subjected to gel electrophoresis (12% bisacrylamide gel) and transferred to nitrocellulose membrane. Blots were blocked in 3% milk Tris-buffered saline Tween and probed with anti-PKC antibody (1 : 500) in 2% milk Tris-buffered saline Tween and probed with an anti-rabbit secondary antibody [20].

For Arg-1 and iNOS expressions, after the treatments, the total protein concentrations were quantified by Bradford reagent. Total protein lysates were subjected to 12% sodium dodecyl SDS-PAGE and transferred onto polyvinylidene difluoride membranes. Membranes were incubated with rabbit anti-mouse primary antibody (anti-iNOS 1 : 1000; anti-Arg-1, 1 : 1000; anti-GAPDH, 1 : 1000) in phosphate buffered saline (PBS) with 0.1% Tween-20 overnight at 4°C and then incubated for 1 h at room temperature with anti-rabbit IgG. GAPDH served as the control. Expression was visualized by enhanced chemiluminescence. Image was assessed by using computerized analysis software (Bio-Rad Laboratories, Hercules, USA).

### 2.4. Immunocytochemistry

The microglial cells were seeded into a confocal microscopy special dish at a density of 2 × 10^4^ cells. Then the microglial cells were divided into five groups: control, A*β*, nicotine (Nico) + A*β*, AM630 + Nico + A*β* and AM630 + A*β* groups. As CB2 receptor antagonist AM630 is lipid soluble, DMSO was used to dissolve AM630. After the dissolution, the DMSO containing AM630 was added into cell culture medium to treat cells, and the final concentration of DMSO was 0.005% (1/20000 in v/v). After the treatments, the cells were fixed with 4% paraformaldehyde solution for 1 h. Then, the cells were blocked with 5% BSA solution after being washed three times with PBS. The cells were incubated at 4°C overnight with the corresponding primary antibody (CB2, 1 : 50; iNOS, 1 : 200; Arg-1, 1 : 200). Then, the cells were incubated in Cy3-labeled (red) or FITC-labeled (green) secondary antibody solution (1 : 200) for 1 h at room temperature. At the end of the incubation, 200 *μ*L of DAPI staining solution was added into the dish. After 5-minute incubation, the dish was washed three times with PBS. Then the dish was observed by using a confocal microscope (FV10i, Olympus, Japan).

### 2.5. Enzyme-Linked Immunosorbent Assay

The supernatants of the cell culture were collected and measured for TNF-*α*, IL-6, IL-10, and BDNF concentrations by using the corresponding quantification ELISA kits according to the manufacturer's instructions. All the experiments were repeated three times. Results are expressed as picograms per litre.

### 2.6. Statistical Analysis

SPSS 13.0 for Windows was used to conduct statistical analysis. All data were expressed as means ± standard deviation (SD). The results were compared by one-way ANOVA, followed by Tukey's Multiple Comparison Test. *P* < 0.05 was considered as statistical significance.

## 3. Results

### 3.1. Nicotine Decreased TNF-*α* and IL-6 Releases in the A*β*-Stimulated N9 Microglia

**Figure 1:**
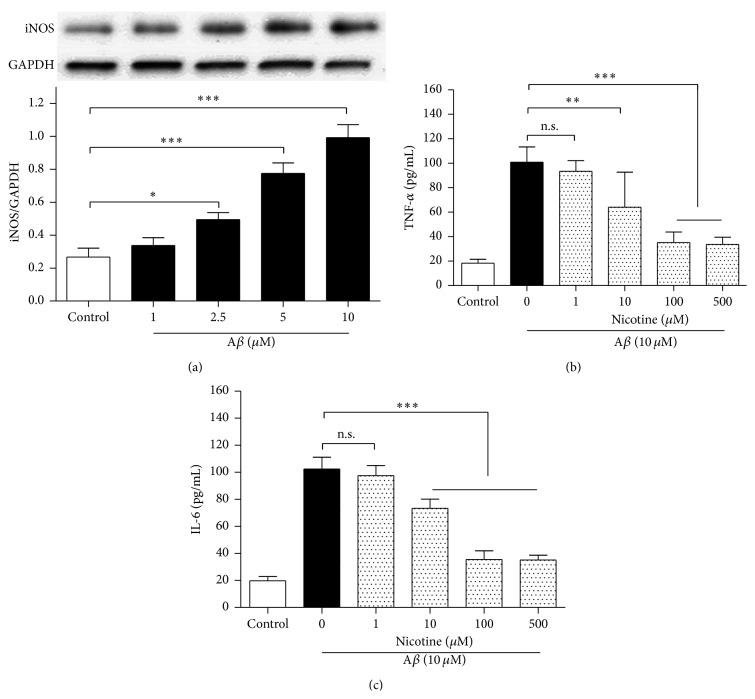
Nicotine decreased TNF-*α* and IL-6 releases from A*β*-stimulated microglial cells in a dose-dependent manner. (a) A*β* exposure increased iNOS expression. The N9 microglial cells were exposed to different concentrations of A*β* for 24 h (*n* = 4). (b) Nicotine decreased TNF-*α* release (*n* = 6). (c) Nicotine decreased IL-6 release (*n* = 6). Results are expressed as means ± SD, ^*∗*^ *P* < 0.05, ^*∗∗*^ *P* < 0.01, ^*∗∗∗*^ *P* < 0.001; n.s.: no significance.

To determine a suitable A*β* concentration, the N9 microglial cells were exposed to the medium containing different concentrations of A*β* for 24 h (Figure 1(a)); then western blot was used to evaluate the expression of inducible nitric oxide synthase (iNOS), a biomarker of microglial activation. The iNOS expression increased dose-dependently in the presence of A*β*, and 5 *μ*M A*β* was used in the subsequent experiments.

Then, the N9 microglial cells were incubated in the medium containing different concentrations of nicotine plus 5 *μ*M A*β*. A*β* exposure increased TNF-*α* and IL-6 concentrations in the medium significantly (Figures 1(b) and 1(c)); treatments of the cells with 10, 100, and 500 *μ*M nicotine, however, reduced the two proinflammatory factors releases obviously (*P* < 0.05), and 100 *μ*M nicotine was taken in the following experiments.

### 3.2. CB2 Receptor Antagonist Reversed Nicotine-Induced Effects on iNOS and Arg-1 Expressions

**Figure 2:**
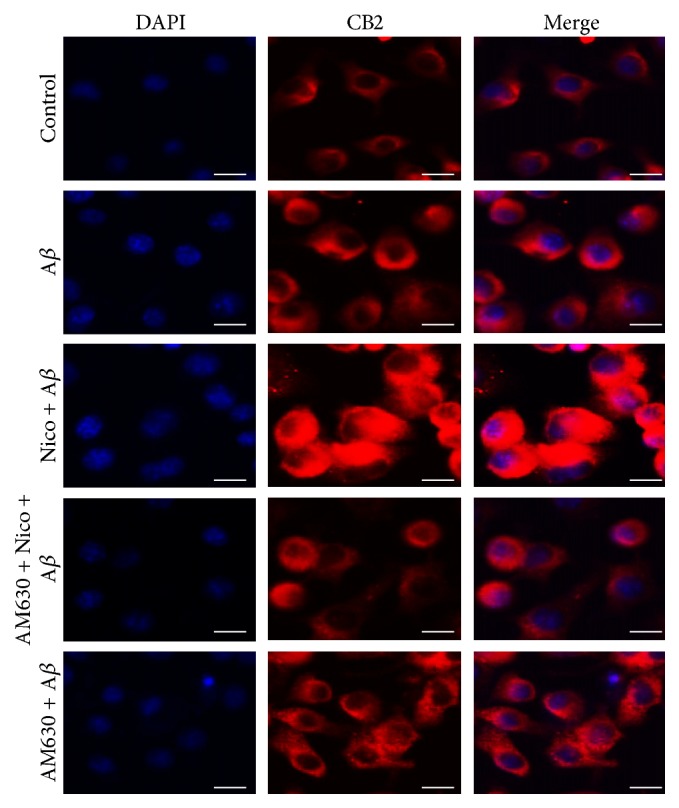
Nicotine increased CB2 receptor expression in microglial cells exposed to A*β*. The microglial cells were divided into five groups: control: cells cultured in drug-free medium; A*β*: cells cultured in the medium containing 5 *μ*M A*β*; nicotine (Nico) + A*β*: cells cultured in the medium containing 10 *μ*M nicotine and 5 *μ*M A*β*; AM630 + Nico + A*β*: cells cultured in the medium containing 10 *μ*M CB2 antagonist AM630, 10 *μ*M nicotine, and 5 *μ*M A*β*; AM630 + A*β*: cells cultured in the medium containing 10 *μ*M AM630 and 5 *μ*M A*β*. After an incubation of 24 h, immunocytochemistry was used in investigating CB2 receptor expression (red), and the nuclei (blue) were counter-stained with DAPI staining solution. Bar = 20 *μ*m.

To investigate nicotine-induced effects on microglial CB2 receptor expression, we used immunocytochemistry (Figure 2). The exposure of 100 *μ*M nicotine for 24 h increased the CB2 protein expression obviously; however, the coadministration of 10 *μ*M AM630, a selective CB2 receptor antagonist, markedly reversed the nicotine-induced upregulation of CB2 expression.

**Figure 3:**
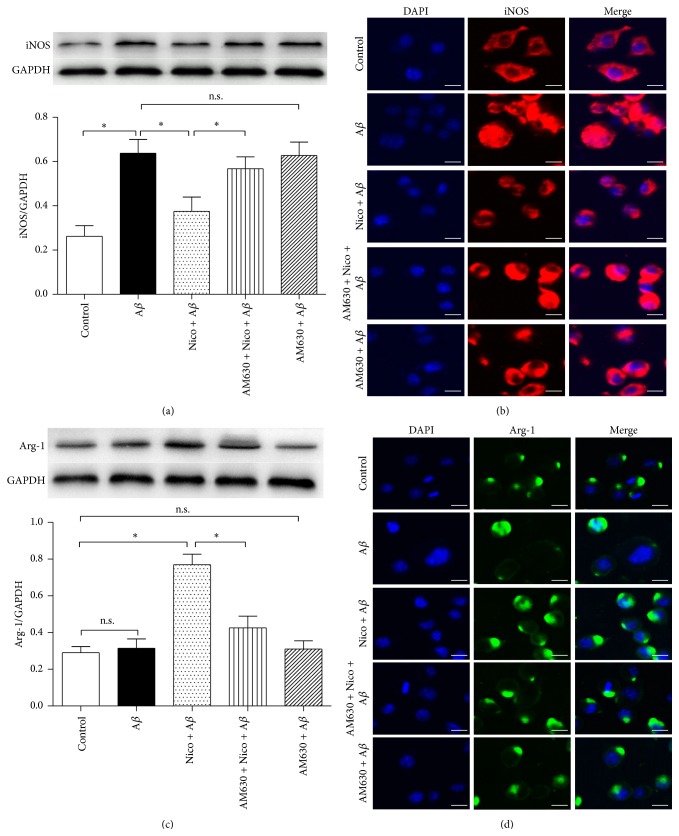
CB2 receptor antagonist reversed nicotine-induced effects on iNOS and Arg-1 protein expressions. The microglial cells were divided into five groups: control: cells cultured in drug-free medium; A*β*: cells cultured in the medium containing 5 *μ*M A*β*; nicotine (Nico) + A*β*: cells cultured in the medium containing 10 *μ*M nicotine and 5 *μ*M A*β*; AM630 + Nico + A*β*: cells cultured in the medium containing 10 *μ*M CB2 antagonist AM630, 10 *μ*M nicotine, and 5 *μ*M A*β*; AM630 + A*β*: cells cultured in the medium containing 10 *μ*M AM630 and 5 *μ*M A*β*. After an incubation of 24 h, western blot (*n* = 4) and immunocytochemistry were used to evaluate iNOS (a-b) and Arg-1 (c-d) expressions. Results are expressed as means ± SD, ^*∗*^ *P* < 0.05; n.s.: no significance. Bar = 10 *μ*m.

The microglial M1 state biomarker iNOS and M2 biomarker Arg-1 expressions were assessed by western blot and immunocytochemistry. Exposure of 100 *μ*M nicotine for 24 h significantly attenuated iNOS expression (Figures 3(a) and 3(b)) and upregulated Arg-1 expression (Figures 3(c) and 3(d)) in the A*β*-treated microglia; 10 *μ*M AM630, nevertheless, partially abolished the nicotine-induced effects on iNOS and Arg-1 expressions (*P* < 0.05). These findings indicated that cannabinoid CB2 receptor may mediate the nicotine-induced modulation of microglial M1/M2 states in the microglial cells exposed to A*β*.

### 3.3. CB2 Receptor Antagonist Reversed Nicotine-Induced Effects on TNF-*α*, IL-6, and BDNF Releases

**Figure 4:**
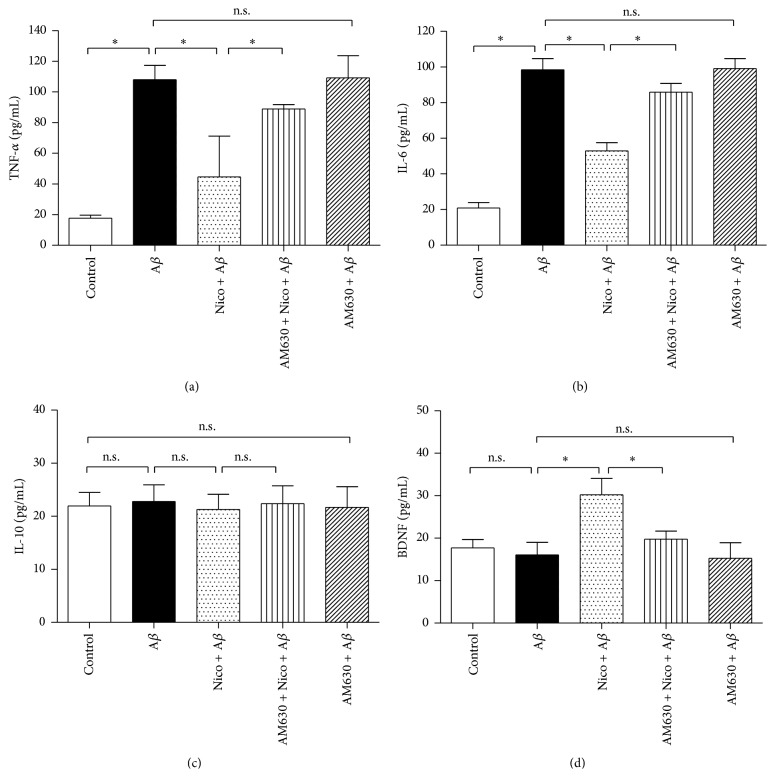
CB2 receptor antagonist partially abolished nicotine-induced effects on TNF-*α*, IL-6, and BDNF releases. The microglial cells were divided into five groups: control: cells cultured in drug-free medium; A*β*: cells cultured in the medium containing 5 *μ*M A*β*; nicotine (Nico) + A*β*: cells cultured in the medium containing 10 *μ*M nicotine and 5 *μ*M A*β*; AM630 + Nico + A*β*: cells cultured in the medium containing 10 *μ*M CB2 antagonist AM630, 10 *μ*M nicotine, and 5 *μ*M A*β*; AM630 + A*β*: cells cultured in the medium containing 10 *μ*M AM630 and 5 *μ*M A*β*. After an incubation of 24 h, concentrations of TNF-*α* (a), IL-6 (b), IL-10 (c), and BDNF (d) in the supernatants were assessed by using the corresponding reagent kit (*n* = 6). Results are expressed as means ± SD, ^*∗*^ *P* < 0.05; n.s.: no significance.

Proinflammatory factors, including TNF-*α* and IL-6, are biomarkers of microglial M1 state; in contrast, anti-inflammatory factor IL-10 and neurotrophic factor BDNF are the biomarkers of microglial M2 state. In this study, compared with the cells treated with A*β* alone, exposure of 100 *μ*M nicotine for 24 h significantly decreased TNF-*α* (Figure 4(a)) and IL-6 (Figure 4(b)) concentrations (*P* < 0.05) and increased BDNF concentration (Figure 4(d)) in the medium (*P* < 0.05); coadministration of 10 *μ*M CB2 antagonist AM630, however, partially reversed the nicotine-induced effects on TNF-*α*, IL-6, and BDNF releases (*P* < 0.05). Interestingly, the concentration of anti-inflammatory IL-10 (Figure 4(c)) remained unchanged (*P* > 0.05). These findings indicated that CB2 receptor may mediate the nicotine-induced effects on the releases of proinflammatory and neurotrophic factors in the N9 microglia exposed to A*β*.

### 3.4. Protein Kinase C Inhibitor Abolished the Nicotine-Induced Anti-Inflammatory Effects

**Figure 5:**
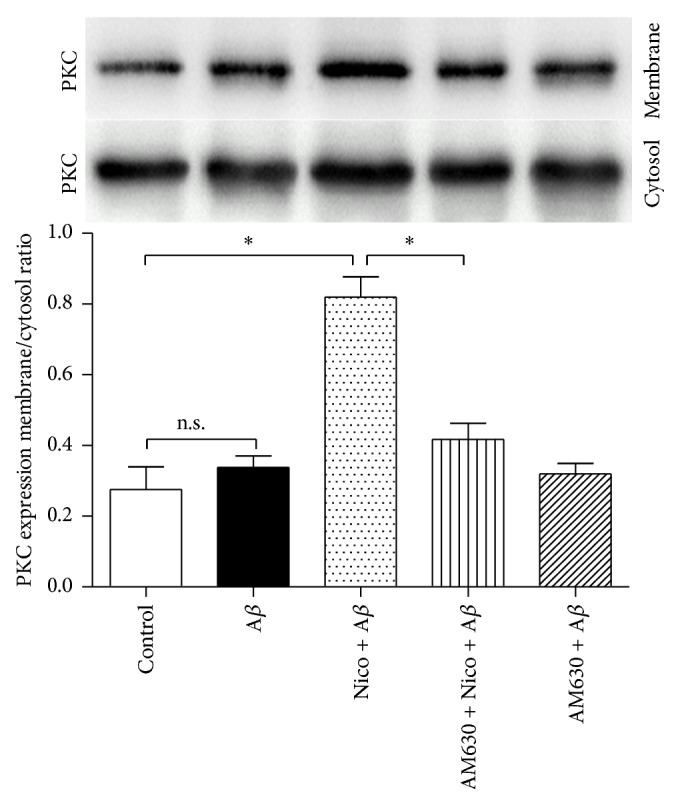
CB2 receptor antagonist abolished nicotine-induced effect on PKC expression. The microglial cells were divided into five groups: control: cells cultured in drug-free medium; A*β*: cells cultured in the medium containing 5 *μ*M A*β*; nicotine (Nico) + A*β*: cells cultured in the medium containing 10 *μ*M nicotine and 5 *μ*M A*β*; AM630 + Nico + A*β*: cells cultured in the medium containing 10 *μ*M CB2 antagonist AM630, 10 *μ*M nicotine, and 5 *μ*M A*β*; AM630 + A*β*: cells cultured in the medium containing 10 *μ*M AM630 and 5 *μ*M A*β*. After an incubation of 24 h, western blot was used to detect PKC expression (*n* = 4). Results are expressed as means ± SD, ^*∗*^ *P* < 0.05; n.s.: no significance.

We also investigated the role of PKC in nicotine-induced anti-inflammations in A*β*-treated microglia. Compared with the cells exposed to 5 *μ*M A*β* alone (Figure 5), 100 *μ*M nicotine increased the expression of PKC in cell membrane significantly (*P* < 0.05), and CB2 receptor antagonist AM630 markedly reversed the nicotine-induced effect on PKC expression (*P* < 0.05).

**Figure 6:**
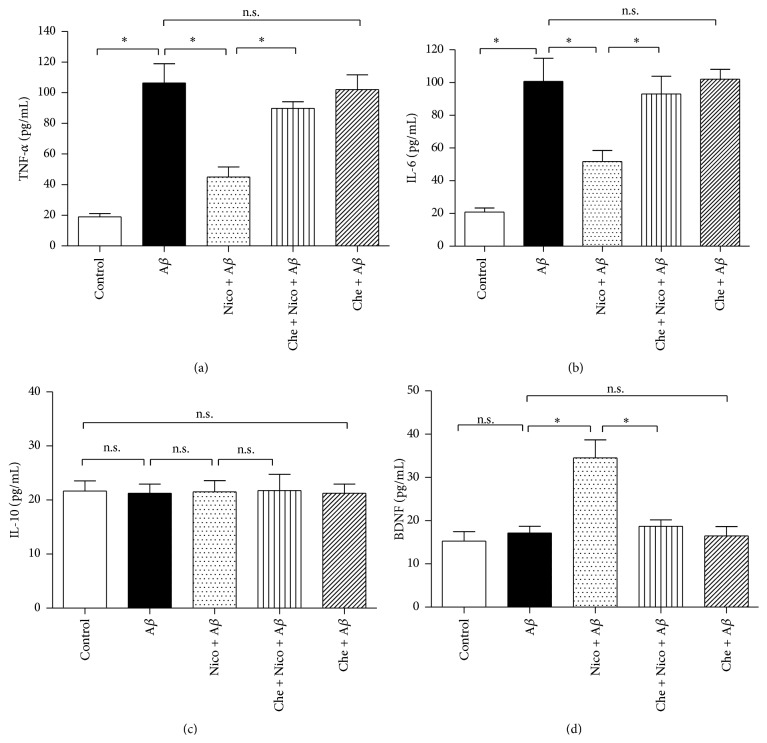
PKC inhibitor reversed nicotine-induced effects on TNF-*α*, IL-6, and BDNF releases. The microglial cells were divided into five groups: control: cells cultured in drug-free medium; A*β*: cells cultured in the medium containing 5 *μ*M A*β*; nicotine (Nico) + A*β*: cells cultured in the medium containing 10 *μ*M nicotine and 5 *μ*M A*β*; Che + Nico + A*β*: cells cultured in the medium containing 10 *μ*M PKC inhibitor chelerythrine (Che), 10 *μ*M nicotine, and 5 *μ*M A*β*; Che + A*β*: cells cultured in the medium containing 10 *μ*M chelerythrine and 5 *μ*M A*β*. After an incubation of 24 h, concentrations of TNF-*α* (a), IL-6 (b), IL-10 (c), and BDNF (d) were assessed by using the corresponding reagent kit (*n* = 6). Results are expressed as means ± SD, ^*∗*^ *P* < 0.05; n.s.: no significance.

Then, we assessed the role of PKC in nicotine-induced effects on cytokines. We found that 100 *μ*M nicotine exposure decreased TNF-*α* and IL-6 releases (Figures 6(a) and 6(b)) and increased BDNF release (Figure 6(d)) from the cells. Coadministration of 10 *μ*M PKC inhibitor chelerythrine (Che) abolished the nicotine-induced benefits significantly (*P* < 0.05). Meanwhile, the IL-10 concentration was still unchanged (Figure 6(c)). These results indicated that PKC may mediate the nicotine-induced anti-inflammations in the A*β*-treated N9 microglia.

## 4. Discussion

In this study, we found that nicotine attenuated A*β*-induced microglial activation, decreased the markers' level of microglial M1 state, including iNOS, TNF-*α*, and IL-6, and increased the markers' level of M2 state, including Arg-1 and BDNF. Coadministration of cannabinoid CB2 antagonist AM630 or PKC inhibitor chelerythrine, however, significantly reversed the nicotine-induced effects on the markers of microglial M1 and M2 states. Our findings indicated that nicotine attenuates A*β*-induced microglial activation by shifting microglial M1 to M2 state via cannabinoid CB2 receptor, and PKC may mediate the process.

Microglial activation is involved in a variety of neurological conditions, including AD, Parkinson's disease (PD), and traumatic brain injury [21–23]. Therefore, inhibiting microglial activation is believed to be an effective therapy for these neurological disorders. Some current investigations indicated that microglia of resting state can be activated into classic activated state (M1 state) or alternative activated state (M2 state) [5–7]. Microglia of M1 state can secrete high levels of proinflammatory factors, which are considered to be harmful; in contrast, microglia of M2 state may produce a variety of anti-inflammatory cytokines and neurotrophins, which are believed to be beneficial. For this reason, shifting microglial M1 to M2 state is regarded to be helpful in treating microglial activation-induced neurological conditions. In brains of AD patients, A*β* deposition is a typical pathological character, which can induce microglial activation and even neuron death [24, 25]. And some recent investigations showed that A*β* can activate microglia into M1 state to secrete inflammatory cytokines [26, 27]. Thus, in this study, we used A*β*-stimulated microglia to mimic the neuroinflammatory response of AD. The mouse N9 microglial cells used in this study, like primary cultured microglia, can be polarized into M1 or M2 state and secrete the markers of microglial M1 and M2 states, such as iNOS, TNF-*α*, IL-6, and Arg-1, in the presence of stimulus. So we used N9 microglia to study microglial M1 and M2 states in this investigation [28, 29]. Nicotine is neuroprotective, and at present, most investigations about nicotine's neuroprotective effects are associated with the activation of nAchR in neuronal cells [12, 13]. However, some studies of nicotine indicated that there might be a cross-talk between nicotine administration and the activation of the endocannabinoid system. For instance, cannabinoid CB2 receptor antagonist is used in treating nicotine addiction [14]. In addition, cannabis THC reduces the incidence of precipitated nicotine withdrawal signs in mice [17], and Chen et al. reported that nicotine attenuates brain ischemia/reperfusion injury in rats via cannabinoid CB1 receptor [30]. Moreover, in a previous study, we found that nicotine protects neurons against A*β* neurotoxicity via CB1 receptor [18]. In this investigation, we found that nicotine inhibited A*β*-induced microglial activation by ameliorating M1/M2 states, leading to the result that less proinflammatory factors and more BDNF were released; CB2 receptor antagonist, nevertheless, partially abolished the nicotine-induced anti-inflammatory and neurotrophic effects. Meanwhile, the level of IL-10, an anti-inflammatory cytokine, did not change significantly; therefore these findings may show that nicotine-induced neuroprotective effects are mainly by decreasing the production of proinflammatory cytokines and increasing the release of neurotrophins, but not by enhancing the secretion of anti-inflammatory factors. Similarly, some studies indicated that nicotine treatment induces neuroprotection by increasing the protein expression level of BDNF in gestational and postnatal rat brain [31, 32]. How does nicotine stimulate CB2 receptor and increase its expression in microglia remains a question to be answered. At present, most studies about the relationship between nicotine administration and cannabinoid system are related to the addiction of nicotine [33–35]. Although nAchRs, including *α*3-, *α*4-, and *α*7-AchRs, are believed to be involved in nicotine-induced bioactivities [36–38], and a study reported that *α*3-nAchR and CB2 receptor were expressed in the same neural cells of brain tissue in mice [39], additional studies are needed to explore the mechanism that nicotine stimulates CB2 receptor and increases its expression.

**Figure 7:**
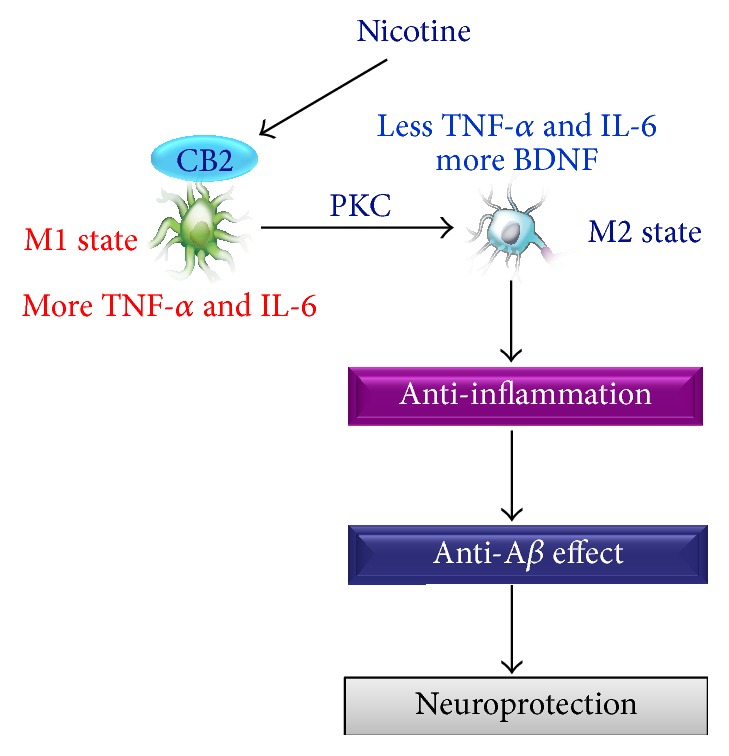
Hypothetical model of CB2 receptor mediates nicotine-induced neuroprotection against A*β* toxicity in microglia. Nicotine exposure increases microglial CB2 receptor expression, which then shifts microglial M1 to M2 state, leading to the result that less TNF-*α* and IL-6 and more BDNF are released from the cells, and the process is mediated by PKC. Then the nicotine-induced anti-inflammation may happen, resulting in anti-A*β* effect and neuroprotection.

PKC is a widely expressed family of serine/threonine kinases. It has been demonstrated that PKC plays a role in neuroprotections against brain ischemic injury [40]. And Xu et al. reported that PKC mediates isoflurane (an inhalable anesthetic) induced anti-inflammation against lipopolysaccharide plus interferon *γ* in microglial cells [41]. In addition, we have ever reported that PKC is involved in nicotine-induced protection against A*β* in neurons [18]. Thus, we investigated the role of PKC in this study. We found that CB2 antagonist AM630 significantly attenuated nicotine-induced upregulation of PKC expression in the cell membrane, and PKC inhibitor chelerythrine markedly abolished the nicotine-induced effects on the secretion of cytokines, suggesting that PKC may mediate nicotine-induced anti-inflammation in N9 microglial cells exposed to A*β*. Similarly, Chen et al. reported that PKC mediated nicotine-induced neuroprotection against A*β* in neurons [42]. PKC is expressed both in cytosol and in cell membrane. PKC can be activated, and after the activation, PKC can translocate from cytosol to cell membrane. Such translocation has been identified to be the hallmark of PKC activation [43]. Cannabinoid CB2 receptor is a G protein-coupled receptor, which is expressed mainly in cell membrane [44]. Therefore, we infer that nicotine activated microglial CB2 receptor and then induced an activation of PKC protein, followed by the decrease of microglial M1 markers, and the increase of microglial M2 markers, resulting in anti-inflammatory and neuroprotective effects (Figure 7).

However, there are still some limitations in our study. First, at present, at least 11 isoforms of PKC have been identified [45], including PKC*α*, PKC*γ*, and PKC*ε*, where isoform of PKC which mediates the nicotine-induced effects in this study has not been investigated. Second, this study is just in vitro; therefore the results of this study should be verified in vivo. In this investigation, we found that cannabinoid CB2 receptor mediated nicotine-induced anti-inflammation against A*β* in microglia by shifting microglial M1 to M2 state via PKC. Our findings of this study offered a novel pharmaceutical target of nicotine and a new therapeutic target for the treatment of AD.

In summary, this study showed that nicotine attenuates A*β*-induced microglial activation by modulating microglial M1/M2 states, and the effects are mediated by cannabinoid CB2 receptor and PKC.

## References

[1] Hardy J., Selkoe D. J. (2002). The amyloid hypothesis of Alzheimer's disease: progress and problems on the road to therapeutics. *Science*.

[2] Akiyama H., Barger S., Barnum S. (2000). Inflammation and Alzheimer's disease. *Neurobiology of Aging*.

[3] Wang S.-W., Wang Y.-J., Su Y.-J. (2012). Rutin inhibits *β*-amyloid aggregation and cytotoxicity, attenuates oxidative stress, and decreases the production of nitric oxide and proinflammatory cytokines. *Neurotoxicology*.

[4] Jamwal S., Singh S., Kaur N., Kumar P. (2015). Protective effect of spermidine against excitotoxic neuronal death induced by quinolinic acid in rats: possible neurotransmitters and neuroinflammatory mechanism. *Neurotoxicity Research*.

[5] Ju L., Zeng H., Chen Y., Wu Y., Wang B., Xu Q. (2015). Dual polarization of microglia isolated from mixed glial cell cultures. *Journal of Neuroscience Research*.

[6] Wehrspaun C. C., Haerty W., Ponting C. P. (2015). Microglia recapitulate a hematopoietic master regulator network in the aging human frontal cortex. *Neurobiology of Aging*.

[7] Benson M. J., Manzanero S., Borges K. (2015). Complex alterations in microglial M1/M2 markers during the development of epilepsy in two mouse models. *Epilepsia*.

[8] McGeer P. L., McGeer E. G. (2015). Targeting microglia for the treatment of Alzheimer’s disease. *Expert Opinion on Therapeutic Targets*.

[9] Araya J. A., Ramírez A. E., Figueroa-Aroca D. (2014). Modulation of neuronal nicotinic receptor by quinolizidine alkaloids causes neuroprotection on a cellular Alzheimer model. *Journal of Alzheimers Disease*.

[10] Liu Q., Zhang J., Zhu H., Qin C., Chen Q., Zhao B. (2007). Dissecting the signaling pathway of nicotine-mediated neuroprotection in a mouse Alzheimer disease model. *The FASEB Journal*.

[11] Ju H. M., Soo Y. K., Hwan G. L., Kim S. U., Yong B. L. (2008). Activation of nicotinic acetylcholine receptor prevents the production of reactive oxygen species in fibrillar *β* amyloid peptide (1–42)-stimulated microglia. *Experimental and Molecular Medicine*.

[12] Marutle A., Gillberg P.-G., Bergfors A. (2013). ^3^H-deprenyl and ^3^H-PIB autoradiography show different laminar distributions of astroglia and fibrillar *β*-amyloid in Alzheimer brain. *Journal of Neuroinflammation*.

[13] Tran M. H., Yamada K., Olariu A., Mizuno M., Ren X. H., Nabeshima T. (2001). Amyloid beta-peptide induces nitric oxide production in rat hippocampus: association with cholinergic dysfunction and amelioration by inducible nitric oxide synthase inhibitors. *The FASEB Journal*.

[14] Gamaleddin I., Zvonok A., Makriyannis A., Goldberg S. R., Le Foll B. (2012). Effects of a selective cannabinoid CB2 agonist and antagonist on intravenous nicotine self administration and reinstatement of nicotine seeking. *PLoS ONE*.

[15] Muldoon P. P., Lichtman A. H., Parsons L. H., Damaj M. I. (2013). The role of fatty acid amide hydrolase inhibition in nicotine reward and dependence. *Life Sciences*.

[16] Gamaleddin I., Wertheim C., Zhu A. Z. X. (2012). Cannabinoid receptor stimulation increases motivation for nicotine and nicotine seeking. *Addiction Biology*.

[17] Balerio G. N., Aso E., Berrendero F., Murtra P., Maldonado R. (2004). Δ9-tetrahydrocannabinol decrease somatic and motivational manifestations of nicotine withdrawal in mice. *European Journal of Neuroscience*.

[18] Wu M., Jia J., Lei C. (2015). Cannabinoid receptor CB1 is involved in nicotine-induced protection against A*β*1-42 neurotoxicity in HT22 cells. *Journal of Molecular Neuroscience*.

[19] Ma L., Jia J., Liu X., Bai F., Wang Q., Xiong L. (2015). Activation of murine microglial N9 cells is attenuated through cannabinoid receptor CB2 signaling. *Biochemical and Biophysical Research Communications*.

[20] Liu W., Dou F., Feng J., Yan Z. (2011). RACK1 is involved in *β*-amyloid impairment of muscarinic regulation of GABAergic transmission. *Neurobiology of Aging*.

[21] Latta C. H., Sudduth T. L., Weekman E. M. (2015). Determining the role of IL-4 induced neuroinflammation in microglial activity and amyloid-*β* using BV2 microglial cells and APP/PS1 transgenic mice. *Journal of Neuroinflammation*.

[22] Koshimori Y., Ko J. H., Mizrahi R. (2015). Imaging striatal microglial activation in patients with Parkinson's disease. *PLoS ONE*.

[23] Wang W., Lu R., Feng D. Y., Liang L., Liu B., Zhang H. (2015). Inhibition of microglial activation contributes to propofol-induced protection against post-cardiac arrest brain injury in rats. *Journal of Neurochemistry*.

[24] Anastasio T. J. (2015). Temporal-logic analysis of microglial phenotypic conversion with exposure to amyloid-*β*. *Molecular BioSystems*.

[25] Wang X., Hu X., Yang Y., Takata T., Sakurai T. (2015). Systemic pyruvate administration markedly reduces neuronal death and cognitive impairment in a rat model of Alzheimer's disease. *Experimental Neurology*.

[26] Mandrekar-Colucci S., Karlo J. C., Landreth G. E. (2012). Mechanisms underlying the rapid peroxisome proliferator-activated receptor-*γ*-mediated amyloid clearance and reversal of cognitive deficits in a murine model of Alzheimer's disease. *The Journal of Neuroscience*.

[27] Salemi J., Obregon D. F., Cobb A. (2011). Flipping the switches: CD40 and CD45 modulation of microglial activation states in HIV associated dementia (HAD). *Molecular Neurodegeneration*.

[28] Tang Y., Li T., Li J. (2014). Jmjd3 is essential for the epigenetic modulation of microglia phenotypes in the immune pathogenesis of Parkinson's disease. *Cell Death and Differentiation*.

[29] Liu H.-C., Zheng M.-H., Du Y.-L. (2012). N9 microglial cells polarized by LPS and IL4 show differential responses to secondary environmental stimuli. *Cellular Immunology*.

[30] Chen Y., Nie H., Tian L. (2013). Nicotine-induced neuroprotection against ischemic injury involves activation of endocannabinoid system in rats. *Neurochemical Research*.

[31] Harrod S. B., Lacy R. T., Zhu J., Hughes B. A., Perna M. K., Brown R. W. (2011). Gestational IV nicotine produces elevated brain-derived neurotrophic factor in the mesocorticolimbic dopamine system of adolescent rat offspring. *Synapse*.

[32] Damborsky J. C., Winzer-Serhan U. H. (2012). Effects of sex and chronic neonatal nicotine treatment on Na^2+^/K^+^/Cl^−^ co-transporter 1, K^+^/Cl^−^ co-transporter 2, brain-derived neurotrophic factor, NMDA receptor subunit 2A and NMDA receptor subunit 2B mRNA expression in the postnatal rat hippocampus. *Neuroscience*.

[33] Gamaleddin I. H., Trigo J. M., Gueye A. B. (2015). Role of the endogenous cannabinoid system in nicotine addiction: novel insights. *Frontiers in Psychiatry*.

[34] Panagis G., Mackey B., Vlachou S. (2014). Cannabinoid regulation of brain reward processing with an emphasis on the role of CB_1_ receptors: a step back into the future. *Frontiers in Psychiatry*.

[35] Gamaleddin I., Guranda M., Scherma M. (2013). AM404 attenuates reinstatement of nicotine seeking induced by nicotine-associated cues and nicotine priming but does not affect nicotine- and food-taking. *Journal of Psychopharmacology*.

[36] Stergiou C., Zisimopoulou P., Tzartos S. J. (2011). Expression of water-soluble, ligand-binding concatameric extracellular domains of the human neuronal nicotinic receptor *α*4 and *β*2 subunits in the yeast *Pichia pastoris*: glycosylation is not required for ligand binding. *The Journal of Biological Chemistry*.

[37] Kuryatov A., Berrettini W., Lindstrom J. (2011). Acetylcholine receptor (AChR) *α*5 subunit variant associated with risk for nicotine dependence and lung cancer reduces (*α*4*β*2) 2*α*5 AChR function. *Molecular Pharmacology*.

[38] Wada T., Naito M., Kenmochi H., Tsuneki H., Sasaoka T. (2007). Chronic nicotine exposure enhances insulin-induced mitogenic signaling via up-regulation of *α*7 nicotinic receptors in isolated rat aortic smooth muscle cells. *Endocrinology*.

[39] Navarrete F., Rodríguez-Arias M., Martín-García E. (2013). Role of CB2 cannabinoid receptors in the rewarding, reinforcing, and physical effects of nicotine. *Neuropsychopharmacology*.

[40] Wang Q., Li X., Chen Y. (2011). Activation of epsilon protein kinase c-mediated anti-apoptosis is involved in rapid tolerance induced by electroacupuncture pretreatment through cannabinoid receptor type 1. *Stroke*.

[41] Xu X., Kim J. A., Zuo Z. (2008). Isoflurane preconditioning reduces mouse microglial activation and injury induced by lipopolysaccharide and interferon-*γ*. *Neuroscience*.

[42] Chen G.-J., Xiong Z., Yan Z. (2013). A*β* impairs nicotinic regulation of inhibitory synaptic transmission and interneuron excitability in prefrontal cortex. *Molecular Neurodegeneration*.

[43] Kraft A. S., Anderson W. B. (1983). Phorbol esters increase the amount of Ca^2+^, phospholipid-dependent protein kinase associated with plasma membrane. *Nature*.

[44] Mnpotra J. S., Qiao Z., Cai J. (2014). Structural basis of G protein-coupled receptor-Gi protein interaction: formation of the cannabinoid CB2 receptor-Gi protein complex. *The Journal of Biological Chemistry*.

[45] Gidday J. M. (2006). Cerebral preconditioning and ischaemic tolerance. *Nature Reviews Neuroscience*.

